# Primary Care Use before Cancer Diagnosis in Adolescents and Young Adults – A Nationwide Register Study

**DOI:** 10.1371/journal.pone.0155933

**Published:** 2016-05-20

**Authors:** Jette Møller Ahrensberg, Morten Fenger-Grøn, Peter Vedsted

**Affiliations:** 1 Research Unit for General Practice, Department of Public Health, Aarhus University, Aarhus, Denmark; 2 Research Centre for Cancer Diagnosis in Primary Care, Department of Public Health, Aarhus University, Aarhus, Denmark; BIDMC, UNITED STATES

## Abstract

**Introduction:**

Survival rates of cancer patients have generally improved in recent years. However, children and older adults seem to have experienced more significant clinical benefits than adolescents and young adults (AYAs). Previous studies suggest a prolonged diagnostic pathway in AYAs, but little is known about their pre-diagnostic healthcare use. This study investigates the use of primary care among AYAs during the two years preceding a cancer diagnosis.

**Methods:**

The study is a retrospective population-based matched cohort study using Danish nationwide registry data. All persons diagnosed with cancer during 2002–2011 in the age group 15–39 years were included (N = 12,306); each participant was matched on gender, age and general practice with 10 randomly selected references (N = 123,060). The use of primary healthcare services (face-to-face contacts, blood tests and psychometric tests) was measured during the two years preceding the diagnosis (index date), and collected data were analysed in a negative binomial regression model.

**Results:**

The cases generally increased their use of primary care already from 8 months before a cancer diagnosis, whereas a similar trend was not found for controls. The increase was observed for all cancer types, but it started at different times: 17 months before a diagnosis of CNS tumour, 12 months before a diagnosis of soft tissue sarcoma, 9 months before a diagnosis of lymphoma, 5–6 months before a diagnosis of leukaemia, bone tumour or GCT, and 3 months before a diagnosis of malignant melanoma.

**Conclusion:**

The use of primary care among AYAs increase several months before a cancer diagnosis. The diagnostic intervals are generally short for malignant melanomas and long for brain tumours. A prolonged diagnostic pathway may indicate non-specific or vague symptomatology and low awareness of cancer among AYAs primary-care personnel. The findings suggest potential of faster cancer diagnosis in AYAs.

## Introduction

Cancer is the second-leading cause of non-accidental death in adolescents and young adults (AYAs) in the western world[1]. The disease accounts for significant morbidity and extensive long-term effects, including increased risk of serious chronic health conditions, hospitalization[2] and impaired mental health[3]. The prognosis has been markedly improved over the past several decades, but some cancer types (e.g. leukaemia) have witnessed more dramatic advances than others (e.g. bone tumour). AYAs have experienced only modest improvements in cancer survival and mortality rates compared with children and older adults, both in general and for most common cancer types[4–6]. Yet, little is known about the reasons for the poorer outcome in AYAs.

Primary care is the first line of contact in many healthcare systems. The general practitioner (GP) plays a central role in preventive healthcare, including screening, diagnosis and treatment. The GP also acts as a gatekeeper between the primary and the secondary healthcare systems and is the first person to turn to for medical assistance.

Rapid diagnosis enables appropriate and timely cancer therapy and increases the chances of cure[7], but prompt diagnosis of cancer remains a substantial challenge. Low incidence, uncharacteristic symptoms, lack of cancer awareness and low healthcare seeking may prevent early detection of cancer in children and AYAs[8]. The GP has a unique role in early cancer diagnosis and must be able to select patients with suspected cancer symptoms for further investigation. Consequently, national fast-track pathways for cancer suspicion have been adopted in several countries, including the UK[9] and Denmark[10]. These initiatives are intended to reduce the time interval from referral to diagnosis and treatment initiation.

The rarity of cancer in young people and the low predictive values of symptoms[8] imply that suspicion of severe illness is sometimes raised exclusively because of changes in the healthcare-seeking behaviour or from clinical intuition[11]. Evidence suggests that AYAs may experience a prolonged diagnostic journey[12,13], but little is known about how and when AYAs start to seek healthcare before a diagnosis of malignant disease. Healthcare-seeking behaviour patterns prior to a cancer diagnosis may provide new knowledge on the ‘diagnostic window’, i.e. the time from first symptom presentation to actual diagnosis[14]. Increased healthcare use can thus be seen as a proxy variable for symptom presentation[15,16]. Previous register studies on healthcare utilisation have shown higher consultation rates for children with cancer. Children who were later diagnosed with a tumour in the central nervous system (CNS) had more consultations during the entire year before the diagnosis[15], sometimes even several years before the diagnosis[17]. The consultation rates also increased from 3 months before a diagnosis of leukaemia and from 5 months before a diagnosis of lymphoma, bone tumour and other solid tumour[15]. These findings suggest potential for reducing the time interval before the diagnosis.

Delay in the diagnosis may have several important consequences for the patient. First, higher mortality, more advanced stages at treatment initiation and stage progression have been reported[17–20]. Second, delays might generate sub-optimal trajectories and low patient satisfaction. Thus, it seems important to explore whether earlier and more expedient diagnosis could be provided. In this study, we aimed to describe the primary healthcare utilisation among AYAs two years before a cancer diagnosis.

## Methods

### Study design and study population

**Fig 1:**
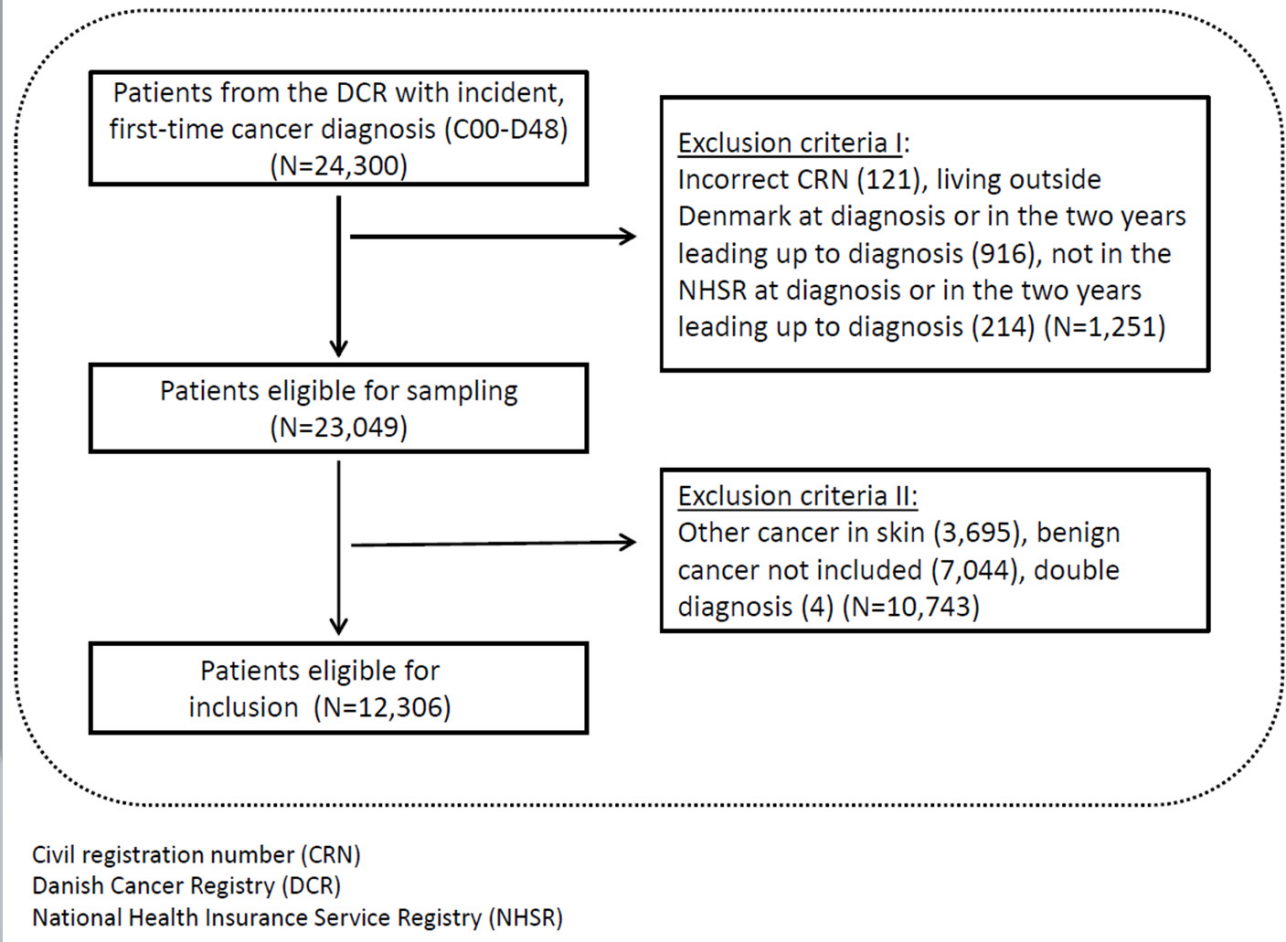
The sampling of patients with incident and primary cancer.

This is a retrospective population-based matched cohort study using information from the Danish Cancer Registry (DCR), the Danish Civil Registration System (CRS) and the Danish National Health Insurance Service Registry (NHSR). We used the civil registration number (CRN), a unique 10-digit personal identification number assigned to every Danish citizen at birth (or immigration), to link information at the individual level in the registers. All included cases and references had a valid CRN, were resident in Denmark and were registered with a general practice in the two-year period leading up to the date of the diagnosis (for cases) or the pseudo-diagnosis (for references), i.e. the date of the cancer diagnosis of their matched case (Fig 1).

All individuals aged 15–39 years with a first-time registration in the DCR during 2002–2011 were included if diagnosed with a malignant ICD-10 code, C00-C97 (except C44 = other cancer in skin), or with an ICD-10 code for a benign tumour in the CNS, D32-33, D35.2–5, D42-43 or D44.3–5 (N = 12,306). The diagnoses were classified according to the Danish version of the International Classification of Diseases (ICD-10)[21] (Fig 1).

In accordance with the ICD-10 codes, all cases were divided into the following nine cancer subtypes: *lymphoma* (C81-85, C88, C90, C96); *germ cell tumour (GCT)* (C56, C62); *CNS tumour* (C70-72, C75.1–3, D32-33, D35.2–4, D42-43, D44.3–5); *malignant melanoma* (C43); *leukaemia* (C91-C95); *bone tumour* (C40-41); *soft tissue tumour* (C45-C49); *unspecified tumour* (C76-C80) and *carcinoma* (all remaining codes of Chapter II in the ICD-10).

Ten references matched on age (+/- 1 year), gender and general practice were randomly selected from the CRS for comparison by using incidence density sampling. A pseudo-diagnosis (index) date was assigned to each of the references. To be eligible for inclusion, matched references were required to be alive and without a history of cancer on the index day.

### Primary healthcare services

The main outcome of the study was consultation rates and diagnostic tests performed in primary care. Information on healthcare utilisation during the 24 months before the diagnosis (index date) was obtained from the NHSR for both cases and references. All primary healthcare services provided to citizens in Denmark are registered prospectively in the NHSR with specific codes, and these data can be linked (through the unique CRN of each individual) to the identification number of the general practice at which the patient is listed. All registrations are based on a fee-for-service remuneration of the health-care provider, and registered records are virtually complete[22]. The NHSR provided data for all consultations and diagnostic tests performed in general practice during the two years before the diagnosis (index date) for all study participants. All daytime face-to-face consultations in primary care (also home visits) were included, except for consultations relating to screening for cervical cancer (smear), pregnancy care and prenatal blood tests. The performed diagnostic procedures comprised both blood tests and psychometric tests. The blood tests included point of care tests performed in primary care (e.g. C-reactive protein, sedimentation rate, differential blood count, haemoglobin and glucose) and tests analysed in hospital laboratories. Examples of psychometric tests were the Major Depression Inventory (MDI) and the Anxiety Symptom Scale (ASS).

### Statistical analyses

For both cases and references, monthly and quarterly (mean) rates with corresponding 95% confidence intervals (CIs) and incidence rate ratios (IRRs) were calculated for face-to-face consultations and diagnostic tests performed in primary care. The 95% CIs were assessed using a negative binomial regression model[23] and applying cluster robust variance estimation to account for dependence between repeated observations on the same subjects. A two-group effect of gender and a linear effect of age (on the implied log scale) were included in the models. Stated rates for consultations and diagnostic tests corresponded to the mean value of the covariates. A priori decided subgroup analyses on face-to-face consultations were performed for each of the nine cancer subtypes: lymphoma, GCT, CNS tumour, malignant melanoma, leukaemia, bone tumour, soft tissue tumour, carcinoma and unspecified tumour. IRRs for blood tests were calculated for the total group of cancer patients, and subgroup analyses were made for haematological cancer (lymphoma and leukaemia), CNS tumour and malignant melanoma. IRRs for the use of psychometric tests were calculated for the total group of cancer patients, and subgroup analyses were made for presence of CNS tumour.

A p-value of 0.05 or less was defined as statistically significant. Data were analysed using Stata 12.0 statistical software (StataCorp LP, TX, USA).

### Ethics statement

The study was approved by the Danish Data Protection Agency (J.no. 2008-41-2956). According to the Committee on Health Research Ethics in the Central Denmark Region, no ethical approval was needed for this study.

## Results

### Characteristics of study participants and reference population

The characteristics of the 12,306 included AYAs diagnosed with cancer and their matched references are shown in Table 1. The mean age of the population was 31.5 years (standard deviation: 6.3 years); 67.7% were aged 30–39 years, and 59.1% were females. In general, men had almost half as many consultations as women (men versus women: 0.49 (95% CI: 0.49–0.50)). The number of consultations rose among women until the age of 30–34 years, whereas consultation rates were more constant among men and showed only minor increase with age (data not shown).

**Table 1:** Characteristics of the 12,306 adolescents and young adult cancer patients.

	Carcinoma		Lymphoma		Germ cell tumour		Central nervous system tumour		Melanoma		Leukaemia		Bone		Soft tissue		Unspecified		Total	
	N	(%)	N	(%)	N	(%)	N	(%)	N	(%)	N	(%)	N	(%)	N	(%)	N	(%)	N	(%)
**All**	4537	(36.9)	765	(6.2)	1837	(14.9)	1569	(12.8)	2501	(20.3)	386	(3.1)	144	(1.2)	315	(2.6)	252	(2.1)	12306	(100.0)
**Gender**																				
Male	834	(16.6)	425	(8.5)	1669	(33.2)	717	(14.3)	785	(15.6)	233	(4.6)	81	(1.6)	143	(2.8)	143	(2.8)	5030	(100.0)
Female	3703	(50.9)	340	(4.7)	168	(2.3)	852	(11.7)	1716	(23.6)	153	(2.1)	63	(0.9)	172	(2.4)	109	(1.5)	7276	(100.0)
**Age**																				
15–19 years	94	(11.8)	136	(17.0)	97	(12.2)	184	(23.1)	93	(11.6)	85	(10.7)	40	(5.0)	36	(4.5)	33	(4.1)	798	(100.0)
20–24 years	205	(17.9)	104	(9.1)	233	(20.4)	165	(14.4)	273	(23.8)	57	(5.0)	43	(3.8)	35	(3.1)	30	(2.6)	1145	(100.0)
25–29 years	542	(26.7)	137	(6.8)	423	(20.9)	277	(13.7)	488	(24.1)	45	(2.2)	22	(1.1)	51	(2.5)	42	(2.1)	2027	(100.0)
30–34 years	1290	(38.5)	161	(4.8)	531	(15.9)	389	(11.6)	718	(21.5)	88	(2.6)	21	(0.6)	80	(2.4)	69	(2.1)	3347	(100.0)
35–39 years	2406	(48.2)	227	(4.6)	553	(11.1)	554	(11.1)	929	(18.6)	111	(2.2)	18	(0.4)	113	(2.3)	78	(1.6)	4989	(100.0)

For each cancer patient, ten references (matched on age, gender and GP) were included.

In the reference population, blood tests were performed twice as often among females than males (Table 2). The use of blood tests was stable among genders; we observed a low tendency towards increased blood test use with age for both genders (men IRR: 1.03, 95% CI: 1.02–1.03, p<0.001); women IRR: 1.02 95% CI: 1.01–1.02, p<0.001) (data not shown).

**Table 2:** Mean rates and corresponding incidence rate ratios (IRR) for consultations and blood tests performed in 3-month intervals in the two years before diagnosis (index date) for AYAs with cancer (cases) and their references (controls).

	Women			Men		
**Consultations**	Cases	References	IRR (95% CI)	Cases	References	IRR (95% CI)
22–24 months	1.04	1.01	1.04 (1.00–1.07)	0.50	0.48	1.04 (0.98–1.11)
19–21 months	1.02	1.00	1.02 (0.99–1.05)	0.50	0.48	1.04 (0.98–1.10)
16–18 months	1.04	1.00	1.04 (1.01–1.07)	0.52	0.48	1.08 (1.02–1.15)
13–15 months	1.06	1.00	1.06 (1.02–1.09)	0.56	0.49	1.14 (1.08–1.21)
10–12 months	1.07	1.00	1.07 (1.04–1.11)	0.56	0.48	1.16 (1.10–1.23)
7–9 months	1.10	1.00	1.09 (1.06–1.13)	0.59	0.49	1.21 (1.14–1.27)
4–6 months	1.22	1.00	1.22 (1.18–1.26)	0.70	0.49	1.44 (1.37–1.52)
1–3 months	2.04	1.00	2.02 (2.00–2.07)	1.80	0.49	3.70 (3.58–3.81)
**Blood tests**	Cases	References	IRR (95% CI)	Cases	References	IRR (95% CI)
22–24 months	0.20	0.20	1.02 (0.93–1.12)	0.09	0.08	1.04 (0.89–1.22)
19–21 months	0.22	0.20	1.13 (1.03–1.22)	0.09	0.08	1.14 (0.97–1.33)
16–18 months	0.22	0.20	1.06 (0.98–1.16)	0.10	0.09	1,10 (0.95–1.28)
13–15 months	0.23	0.21	1.12 (1.03–1.22)	0.11	0.09	1.23 (1.06–1.42)
10–12 months	0.23	0.21	1.12 (1.03–1.21)	0.11	0.09	1.20 (1.03–1.40)
7–9 months	0.25	0.21	1.18 (1.08–1.28)	0.15	0.09	1.58 (1.37–1.82)
4–6 months	0.29	0.21	1.37 (1.26–1.48)	0.16	0.10	1.71 (1.50–1.96)
1–3 months	0.52	0.21	2.34 (2.19–2.51)	0.61	0.10	6.35 (5.82–6.93)

### Face-to-face consultations in general practice

**Fig 2:**
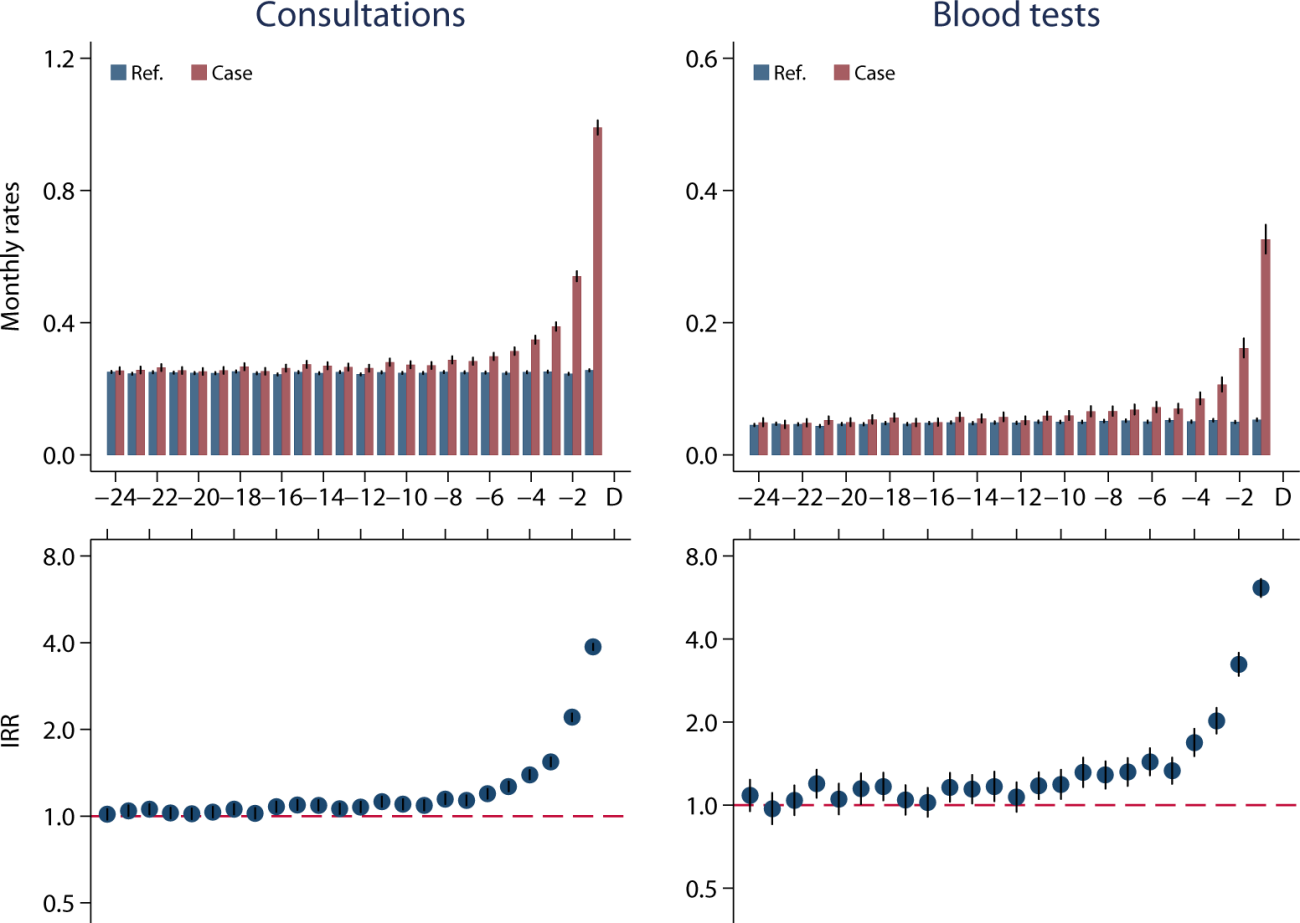
Upper part: Mean rates for consultations and blood tests in primary care for cases and references two years before the diagnosis (index date). Note the difference in the Y-axis range. Lower part: incidence rate ratios (IRR) for consultations with 95% confidence intervals.

Cases and references of both genders had very similar numbers of consultations in primary care until 8 months before the date of diagnosis (index date). A progressive increase in consultations was then observed for the cases (Fig 2), especially during the last three months prior to cancer diagnosis (women: IRR: 2.02, 95% CI: 2.00–2.07, p<0.001; men: IRR: 3.70, 95% CI: 3.58–3.81, p<0.001) (Table 2).

**Fig 3:**
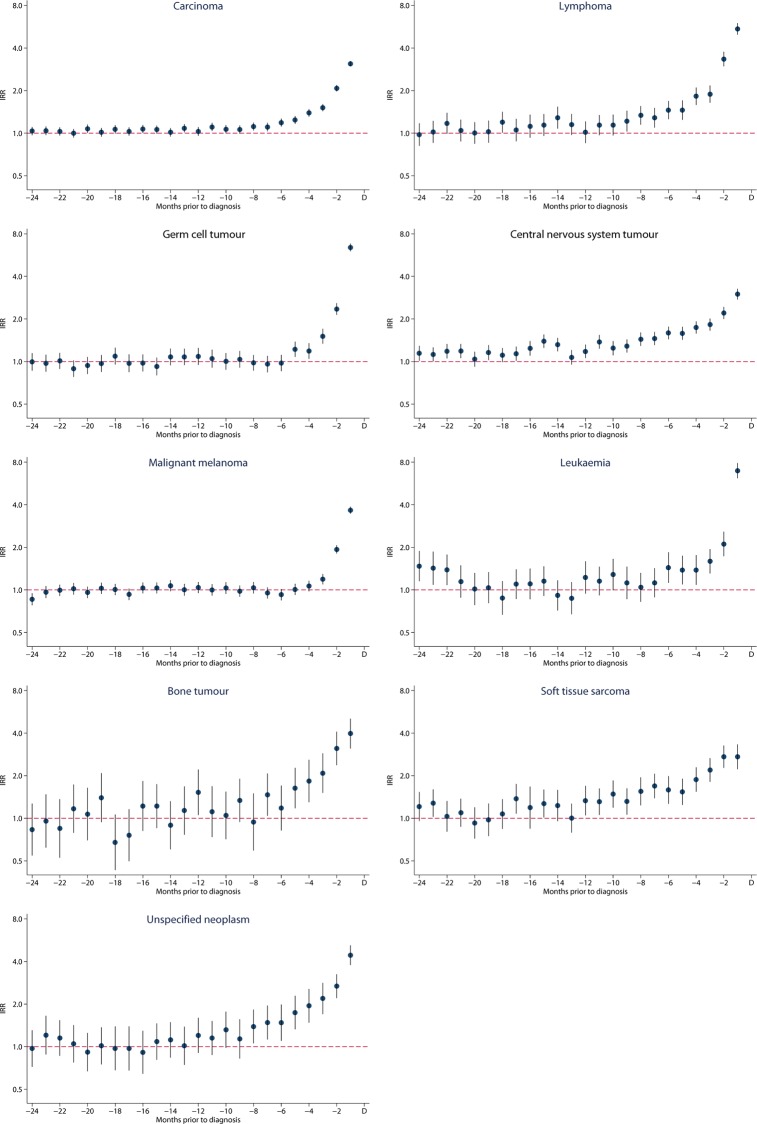
Incidence rate ratios (IRR) for consultations in primary care with 95% confidence intervals for carcinoma, lymphoma, germ cell tumour, central nervous system tumour, malignant melanoma, leukaemia bone tumour, soft tissue tumour and unspecified neoplasm two years before diagnosis (index date).

A minor increase in consultations among AYAs who were subsequently diagnosed with cancer was observed already from 16 months before the diagnosis (IRR: 1.08, 95% CI: 1.03–1.12, p<0.001) (data in S1 Table). The increase in GP attendance was seen for all cancer types, but it started at different time points before the diagnosis: 17 months for CNS tumours, 12 months for soft tissue sarcomas, 9 months for lymphomas, 5–6 months for leukaemia, bone tumours and GCT, and 3 months before diagnosis for malignant melanomas (Fig 3) (data in S1 Table).

In total, 14.9% (1,831) of all included cases (12,306) had more than three consultations during the 1–3 months before diagnosis, whereas the corresponding percentage was 4.0% (4,911) for the included references (123,060).

### Diagnostic tests in primary care

The AYAs who were subsequently diagnosed with cancer had more blood tests performed in primary care than the references from 11 months before the diagnosis (Fig 2) (data in S2 Table). This tendency was particularly marked for males in the last 3 months before a cancer diagnosis (IRR: 6.35, 95% CI: 5.82–6.93) (P<0.001). The relative increase was less substantial for women; no more than 2.34 (IRR: 2.34, 95% CI: 2.19–2.51, p<0.001) in the last 1–3 months prior to a cancer diagnosis (Table 2).

**Fig 4:**
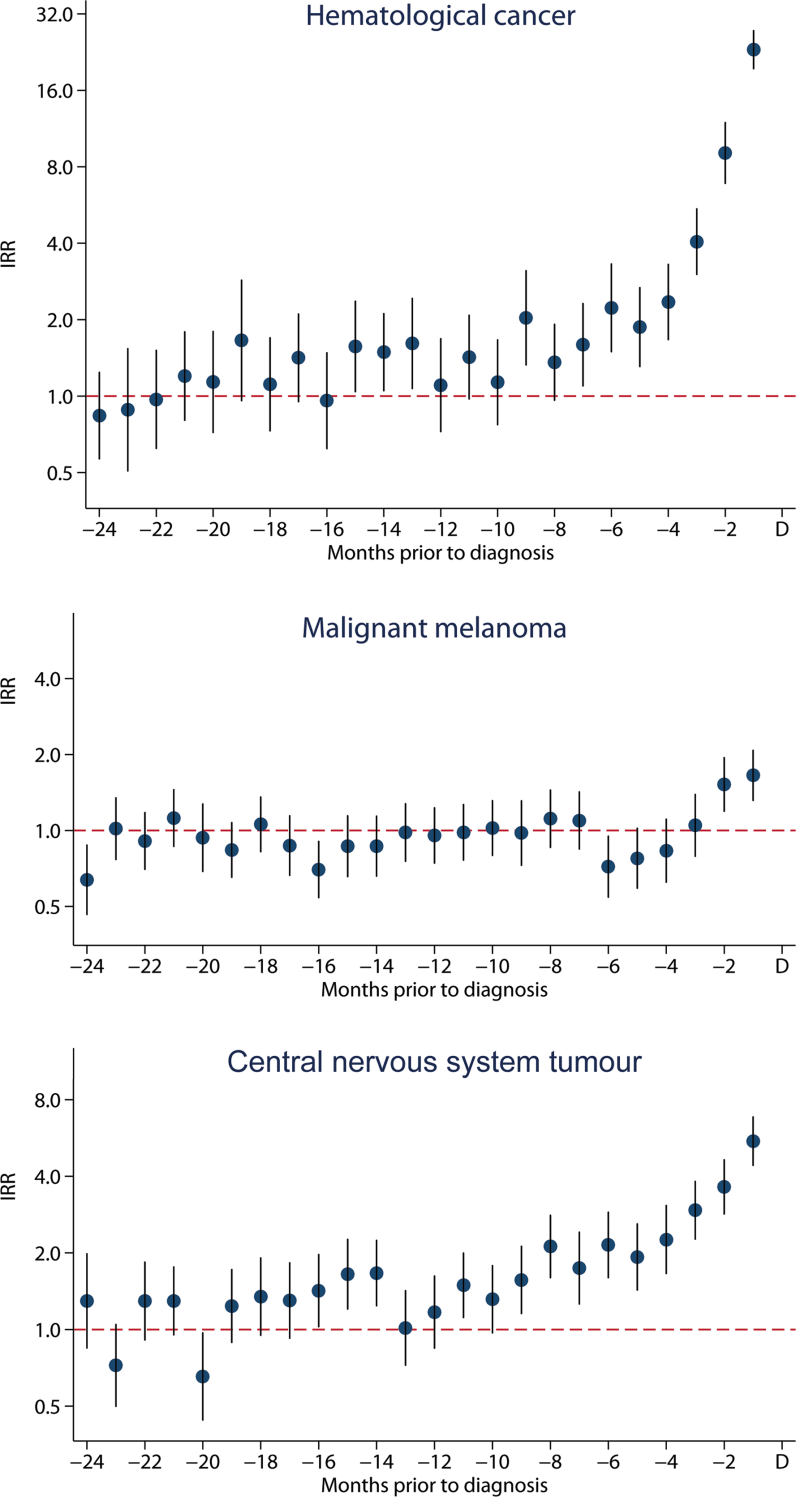
Incidence rate ratios (IRR) for blood tests in primary care with 95% confidence intervals for haematological cancer (lymphoma and leukaemia), malignant melanoma and central nervous system tumour two years before diagnosis (index date).

The increase in blood tests was observed at different time points before the diagnosis, depending on cancer type; 7 months before a diagnosis of haematological cancer (leukaemia and lymphoma), 9 months before a CNS tumour and 2 months before a diagnosis of malignant melanoma (Fig 4) (data in S2 Table).

Except for a minor increase (IRR: 1.49, 95% CI: 1.05–2.10, p = 0.024) in the last month before the date of the diagnosis, no significant general increase was observed in the use of psychometric tests for AYAs (data in S3 Table). However, AYAs who were subsequently diagnosed with a brain tumour had more tests performed than their references in the last 6 months before the diagnosis; twice as many tests were performed in the 4–6 months before the diagnosis (IRR: 2.57, 95% CI: 1.45–4.54, p = 0.001), and three times as many tests were performed in the 1–3 months before the diagnosis (IRR: 3.14, 95% CI: 1.75–5.64, p<0.001).

## Discussion

### Main findings

This large population-based study showed an increased use of healthcare services in primary care in the time period leading up to the cancer diagnosis in AYAs. More consultations were observed already from 16 months before a cancer diagnosis, but the healthcare-seeking rates increased exponentially from 8 months before the final diagnosis. The increase was observed for all cancer types, but the timing of onset depended on cancer type. AYAs with leukaemia, a bone tumour or a GCT had more frequent healthcare requests from 6 months before the diagnosis, whereas the corresponding time point for AYAs with a brain tumour was 17 months before the diagnosis. AYAs with malignant melanoma had more consultations from 3 months before the diagnosis.

Primary healthcare services can be seen as a proxy for symptom presentation, including an indicator of a patient´s degree of discomfort or concern. The identified healthcare seeking patterns suggest that some cancer types in AYAs are difficult to diagnose and that the diagnostic window for some tumours, e.g. brain tumours and soft tissue sarcomas, is relatively broad.

Our study shows an increase in performed blood tests in primary care already from 11 months before a cancer diagnosis. These results indicate that diagnostic workup is performed in primary care although the patient-reported symptoms may not directly point towards malignant disease. Further, we found that more psychometric tests were performed during the 6 months prior to the diagnosis of a CNS tumour. This may suggest that some young cancer patients present with cognitive symptoms; a finding which is in line with a previous Danish study[24].

### Strengths and weaknesses

Information on cancer diagnosis in AYAs was registered prospectively in the Danish Cancer Registry. All recorded diagnoses were based on the WHO International Classification of Diseases (ICD) and coded by the physician responsible for the discharge. The diagnostic records in the register have formerly been shown to be accurate and complete[25]. References were randomly selected among all individuals in Denmark, and these were matched on age, gender and GP with the included cases. We obtained complete information on prospectively recorded primary healthcare use for all AYAs diagnosed with cancer in Denmark during a ten-year period and for a random sample of references of the same age and gender. As the provided consultations and the diagnostic procedures are coded for remuneration purposes, both the accuracy and the completeness of data in the NHSR are high. Selection and information bias in relation to classification of diagnosis and healthcare services are unlikely to explain the results in our study.

In a previous study on childhood cancer patients, we found increased utilisation of primary care services from six months before diagnosis[15]. However, for children with CNS tumours, a higher consultation rate was seen in all 12 months before the diagnosis[15]. Consequently, we considered a 2-year period appropriate in this study of AYAs.

Although cancer in AYAs is rare, we obtained sufficient data to ensure relatively high statistical precision. This allowed us to detect small, but clinically relevant, differences between the groups. We eliminated potential confounding effects of age and gender by matching included cases with references. Consultations related to pregnancy care, cancer screening and healthcare control for chronical disease have specific codes, which were not included in the study. Increased number of visits among cases was considered to be symptom-related, but a few of the included consultations might not have been entirely problem-oriented. This potential confounding was reduced by using a large dataset and matching cases on age, gender and GP. By matching on GP, we reduced the area-specific social differences in the population. However, we cannot exclude that residual confounding by other factors, such as ethnicity, might have played a role. Nevertheless, the vast majority of the Danish population is Caucasians, and ethnicity is rarely among the main concerns in Danish studies. We had no information on pre-existing comorbidity, but this is considered of little importance as comorbidity is relatively infrequent in young people.

The nationwide approach allows us to consider the results as generalizable. Our findings are likely to be applicable in countries with similar healthcare systems providing access for patients through primary care.

### Comparison with other studies

A key area for improving cancer care in several countries has been reducing the time to diagnosis[10,26]. Cancer care in Denmark and the UK has received increased attention in recent years because of low survival rates and advanced tumour stages at treatment initiation compared to the rest of Western Europe[27]. Consultation requests among children with a brain tumour has previously shown to increase 1–2 years before the diagnosis[15,17]. Furthermore, long intervals with symptoms (up to 60 months) are generally reported before a diagnosis of soft tissue sarcomas in children and adolescents[28]. Nevertheless, only few studies have focused on delays in the diagnosis and the pre-diagnostic healthcare utilisation among AYAs.

A large British study showed that cancer patients aged 16–25 years were twice as likely as older adults to have three or more GP consultations before referral for further diagnostic procedures[29]. Additionally, a survey among teenagers and young adults treated for cancer showed that 57% visited their GP three times or more before being referred to a hospital in the time leading up to the diagnosis. Of these, 29% visited their GP more than five times before referral[12]; this suggests a prolonged diagnostic pathway among AYAs.

Several studies have explored the time intervals between diagnosis and treatment of childhood or adult cancer. Our study indicates a longer overall delay among AYAs than observed among children[15,30], but the intervals tend to vary among different cancer types and age groups at diagnosis[13]. Younger adults with bone cancer were more likely to have prolonged symptomatic intervals and more patient delays than children[31]. AYAs also seem to have a prolonged diagnostic pathway compared to older adults. In a large population-based Danish study[14], a modest rise in primary care consultations was observed five to six months before diagnosis, which is two to three months closer to the diagnosis than seen in our study.

In line with previous studies, we observed patterns indicating a relatively broad diagnostic window for some groups of cancer patients. For example, such window was found for AYAs who were subsequently diagnosed with a brain tumour or soft tissue sarcoma, whereas other cancer patients (e.g. patients with malignant melanoma) are generally referred and diagnosed shortly after symptom presentation. Our findings demonstrate that the diagnosis of certain types of cancer, specifically in the group of AYAs, is extremely complex as the ‘symptom signature’ tends to be unspecific. In line with our findings, the odds ratio for experiencing a long diagnostic interval (from first clinical presentation until diagnosis) has been reported to be four times higher in adult cancer patients presenting with vague and uncharacteristic symptoms than in patients presenting with ‘alarm’ or ‘serious’ symptoms[18].

In our study, almost twice as many consultations were seen among young women than among young men. This is not surprising as the consultation pattern among women generally seems to increase until the age of the mid-30s. This pattern is generally known and corresponds very well the general picture in Denmark[32].

### Conclusion and implications

Cancer survival has increased in recent years. However, AYAs do not seem to have benefitted from this development as much as other patient groups. Although cancer is relatively uncommon among AYAs, it is crucial to ensure that the most prevalent cancer types in this patient group are diagnosed without delay.

This study demonstrates a marked increase in both health-care seeking and clinical activity in primary care several months before a cancer diagnosis. This finding indicates that the symptom interpretation starts long before the diagnosis, specifically some types of cancer, in AYAs. The study also stresses the importance of recognising even vague symptoms or minor changes in the help-seeking patterns seen in primary care.

Identifying the few patients with malignant cancer disease is a major challenge in primary care, and the knowledge of symptoms that may indicate early signs of cancer seems to remain sparse in clinical practice. These issues must be addressed if we aim to reduce the diagnostic delays.

Diagnosing cancer in this age group may be particularly difficult because of vague symptoms, but also because of their physical, emotional, cognitive and social development. Furthermore, the cancer incidence is low, and most symptomatic patients have non-malignant illnesses. In addition, low awareness of cancer in this age group may also partly explain the complexity of this field.

Further research is needed to identify the characteristics of early symptoms and optimal diagnostic pathways for AYAs, particularly for the groups with the longest pre-diagnostic interval. Such knowledge may facilitate early identification of patients at increased risk of experiencing a prolonged diagnostic delay.

## Supporting Information

S1 Table Incidence rate ratios (IRR) for consultations in primary care with 95% confidence intervals for carcinoma, lymphoma, germ cell tumour, central nervous system tumour, malignant melanoma, leukaemia bone tumour, soft tissue tumour, unspecified neoplasm and the total group two years before diagnosis (index date). (DOCX)

S2 Table Incidence rate ratios (IRR) for blood tests in primary care with 95% confidence intervals for haematological cancer (lymphoma and leukaemia), malignant melanoma, central nervous system tumour and the total group two years before diagnosis (index date). (DOCX)

S3 Table Incidence rate ratios (IRR) for psychometric tests in primary care with 95% confidence intervals for central nervous system tumour and the total group two years before diagnosis (index date). (DOCX)
