# Supplementary material for: Primary Care Use before Cancer Diagnosis in Adolescents and Young Adults – A Nationwide Register Study
Source: PLoS One. 2016 May 20;11(5):e0155933. doi: 10.1371/journal.pone.0155933 (PMC4874574; doi:10.1371/journal.pone.0155933)
Supplement: S1 Table — (DOCX) [file pone.0155933.s001.docx]

**S1.** *Incidence rate ratios (IRR) for consultations in primary care with 95% confidence intervals for carcinoma, lymphoma, germ cell tumour, central nervous system tumour, malignant melanoma, leukaemia* *bone tumour, soft tissue tumour, unspecified neoplasm and the total group two years before diagnosis (index date)*

|  | **Carcinoma** | **Lymphoma** | **Germ cell tumour** | **Central nervous system tumour** | **Melanoma** | **Leukaemia** | **Bone** | **Soft tissue** | **Unspecified** | **Total** |
| --- | --- | --- | --- | --- | --- | --- | --- | --- | --- | --- |

| **Months before diagnosis** | IRR (95%CI) | IRR (95%CI) | IRR (95%CI) | IRR (95%CI) | IRR (95%CI) | IRR (95%CI) | IRR (95%CI) | IRR (95%CI) | IRR (95%CI) | IRR (95%CI) |
| --- | --- | --- | --- | --- | --- | --- | --- | --- | --- | --- |
| 24 | 1.03(0.97-1.10) | 0.98(0.82-1.17) | 0.99(0.86-1.14) | 1.14(1.01-1.29) | 0.86(0.78-0.94) | 1.47(1.16-1.88) | 0.83(0.55-1.27) | 1.21(0.96-1.53) | 0.97(0.72-1.30) | 1.01(0.97-1.06) |
| 23 | 1.04(0.97-1.11) | 1.02(0.86-1.22) | 0.97(0.85-1.11) | 1.12(1.01-1.26) | 0.97(0.88-1.06) | 1.42(1.09-1.86) | 0.96(0.62-1.47) | 1.28(1.03-1.60) | 1.21(0.88-1.65) | 1.04(1.00-1.09) |
| 22 | 1.03(0.97-1.10) | 1.17(0.99-1.39) | 1.01(0.89-1.15) | 1.18(1.06-1.32) | 0.99(0.91-1.09) | 1.39(1.09-1.77) | 0.85(0.53-1.36) | 1.03(0.81-1.32) | 1.15(0.86-1.54) | 1.06(1.01-1.10) |
| 21 | 1.00(0.93-1.07) | 1.04(0.88-1.24) | 0.89(0.78-1.02) | 1.19(1.06-1.33) | 1.02(0.93-1.12) | 1.15(0.88-1.49) | 1.17(0.79-1.73) | 1.09(0.87-1.37) | 1.05(0.77-1.42) | 1.03(0.98-1.07) |
| 20 | 1.07(1.01-1.15) | 1.00(0.84-1.19) | 0.94(0.82-1.07) | 1.04(0.92-1.17) | 0.96(0.88-1.05) | 1.02(0.78-1.32) | 1.07(0.70-1.64) | 0.93(0.72-1.19) | 0.91(0.67-1.25) | 1.02(.098-1.06) |
| 19 | 1.01(0.95-1.09) | 1.03(0.86-1.22) | 0.97(0.85-1.11) | 1.16(1.03-1.30) | 1.03(0.94-1.12) | 1.04(0.81-1.33) | 1.40(0.94-2.08) | 0.97(0.75-1.27) | 1.01(0.75-1.37) | 1.03(0.99-1.08) |
| 18 | 1.06(1.00-1.14) | 1.19(1.01-1.41) | 1.09(0.95-1.25) | 1.11(0.99-1.24) | 1.01(0.92-1.10) | 0.88(0.67-1.15) | 0.68(0.43-1.06) | 1.07(0.84-1.36) | 0.97(0.68-1.39) | 1.06(1.01-1.10) |
| 17 | 1.03(1.00-1.14) | 1.05(0.88-1.26) | 0.97(0.84-1.12) | 1.14(1.02-1.27) | 0.93(0.85-1.02) | 1.10(0.87-1.39) | 0.76(0.50-1.16) | 1.37(1.08-1.74) | 0.97(0.68-1.39) | 1.02(0.98-1.07) |
| 16 | 1.07(1.00-1.14) | 1.12(0.93-1.35) | 0.98(0.85-1.12) | 1.24(1.10-1.39) | 1.03(0.95-1.13) | 1.10(0.86-1.41) | 1.22(0.82-1.83) | 1.19(0.85-1.67) | 0.91(0.64-1.29) | 1.08(1.03-1.12) |
| 15 | 1.06(0.99-1.13) | 1.14(0.96-1.36) | 0.92(0.80-1.06) | 1.39(1.25-1.55) | 1.03(0.95-1.13) | 1.15(0.91-1.46) | 1.22(0.85-1.74) | 1.27(1.02-1.59) | 1.08(0.81-1.45) | 1.09(1.05-1.14) |
| 14 | 1.01(0.95-1.08) | 1.29(1.08-1.53) | 1.08(0.94-1.23) | 1.32(1.18-1.46) | 1.07(0.98-1.17) | 0.91(0.72-1.17) | 0.89(0.60-1.32) | 1.23(0.96-1.58) | 1.12(0.84-1.49) | 1.09(1.05-1,14) |
| 13 | 1.06(0.99-1.13) | 1.15(0.97-1.36) | 1.08(0.95-1.23) | 1.07(0.95-1.20) | 1.00(0.91-1.10) | 0.87(0.67-1.13) | 1.13(0.77-1.68) | 1.00(0.79-1.26) | 1.01(0.75-1.38) | 1.06(1.02-1.11) |
| 12 | 1.03(0.96-1.10) | 1.02(0.85-1.21) | 1.09(0.95-1.24) | 1.18(1.06-1.31) | 1.03(0.94-1.13) | 1.23(0.94-1.59) | 1.53(1.06-2.20) | 1.33(1.05-1.69) | 1.20(0.91-1.59) | 1.08(1.03-1.12) |
| 11 | 1.11(1.05-1.18) | 1.14(0.97-1.34) | 1.05(0.91-1.21) | 1.38(1.24-1.54) | 0.98(0.90-1.07) | 1.16(0.92-1.46) | 1.11(0.74-1.68) | 1.31(1.06-1.62) | 1.15(0.88-1.51) | 1.12(1.08-1.17) |
| 10 | 1.07(1.00-1.14) | 1.14(0.97-1.35) | 1.00(0.88-1.14) | 1.24(1.11-1.39) | 1.04(0.95-1.13) | 1.28(1.00-1.65) | 1.05(0.71-1.54) | 1.48(1.19-1.84) | 1.32(0.98-1.76) | 1.10(1.05-1.14) |
| 9 | 1.06(0.99-1.13) | 1.22(1.03-1.44) | 1.04(0.91-1.18) | 1.29(1.16-1.43) | 1.00(0.91-1.09) | 1.12(0.86-1.46) | 1.34(0.94-1.90) | 1.31(1.06-1.62) | 1.14(0.83-1.56) | 1.09(1.05-1.14) |
| 8 | 1.11(1.05-1.18) | 1.34(1.15-1.55) | 0.98(0.86-1.11) | 1.44(1.29-1.60) | 1.03(0.94-1.13) | 1.04(0.83-1.32) | 0.94(0.59-1.49) | 1.55(1.24-1.94) | 1.39(1.06-1.82) | 1.15(1.10-1.19) |
| 7 | 1.11(1.03-1.18) | 1.29(1.10-1.50) | 0.96(0.84-1.09) | 1.45(1.31-1.62) | 0.95(0.87-1.04) | 1.12(0.89-1.42) | 1.47(1.04-2.07) | 1.69(1.39-2.06) | 1.48(1.13-1.95) | 1.13(1.09-1.18) |
| 6 | 1.19(1.12-1.26) | 1.46(1.26-1.68) | 0.98(0.86-1.11) | 1.59(1.44-1.76) | 0.92(0.85-1.01) | 1.44(1.13-1.84) | 1.18(0.82-1.70) | 1.59(1.27-1.98) | 1.48(1.10-1.98) | 1.20(1.15-1.24) |
| 5 | 1.24(1.17-1.32) | 1.46(1.25-1.70) | 1.22(1.08-1.38) | 1.58(1.43-1.76) | 1.01(0.92-1.10) | 1.38(1.10-1.74) | 1.64(1.18-2.27) | 1.54(1.25-1.90) | 1.74(1.33-2.28) | 1.27(1.22-1.32) |
| 4 | 1.39(1.31-1.48) | 1.82(1.59-2.10) | 1.19(1.05-.134) | 1.74(1.58-1.91) | 1.06(0.98-1.16) | 1.38(1.09-1.76) | 1.83(1.30-2.58) | 1.88(1.55-2.29) | 1.94(1.48-2.55) | 1.39(1.34-1.45) |
| 3 | 1.52(1.43-1.60) | 1.89(1.65-2.17) | 1.51(1.34-1.70) | 1.82(1.66-2.01) | 1.19(1.10-1.29) | 1.59(1.31-1.94) | 2.09(1.52-2.87) | 2.19(1.82-2.65) | 2.19(1.70-2.81) | 1.54(1.49-1,60) |
| 2 | 2.08(1.98-2.18) | 3.34(2.98-3.75) | 2.35(2.15-2.58) | 2.21(2.01-2.43) | 1.94(1.81-2.07) | 2.11(1.73-2.57) | 3.12(2.38-4.08) | 2.73(2.28-3.26) | 2.68(2.21-3.24) | 2.20(2.14-2.27) |
| 1 | 3.10(2.98-3.22) | 5.46(4.98-5.98) | 6.41(6.04-6.80) | 3.00(2.75-3.26) | 3.66(3.47-3.87) | 6.97(6.18-7.86) | 3.97(3.12-5.05) | 2.72(2.23-3.32) | 4.43(3.79-5.19) | 3.87(3.77-3.97) |
