# Supplementary material for: Primary Care Use before Cancer Diagnosis in Adolescents and Young Adults – A Nationwide Register Study
Source: PLoS One. 2016 May 20;11(5):e0155933. doi: 10.1371/journal.pone.0155933 (PMC4874574; doi:10.1371/journal.pone.0155933)
Supplement: S2 Table — (DOCX) [file pone.0155933.s002.docx]

**S2.** *Incidence rate ratios (IRR) for blood tests in primary care with 95% confidence intervals for haematological cancer (lymphoma and leukaemia), malignant melanoma, central nervous system tumour and the total group two years before diagnosis (index date)*

|  | **Hematological cancer** | **Malignant melanoma** | **Central nervous system tumour** | **Total** |
| --- | --- | --- | --- | --- |
| **Months before diagnosis** | IRR (95%CI) | IRR (95%CI) | IRR (95%CI) | IRR (95%CI) |
| 24 | 0.84(0.57-1.24) | 0.64(0.46-0.87) | 1.30(0.85-1.98) | 1.08(0.95-1.24) |
| 23 | 0.88(0.51-1.54) | 1.02(0.77-1.35) | 0.72(0.50-1.05) | 0.97(0.85-1.11) |
| 22 | 0.97(0.62-1.51) | 0.91(0.70-1.18) | 1.29(0.91-1.84) | 1.04(0.92-1.18) |
| 21 | 1.20(0.80-1.79) | 1.12(0.86-1.45) | 1.30(0.95-1.76) | 1.20(1.06-1.34) |
| 20 | 1.14(0.72-1.80) | 0.94(0.69-1.27) | 0.65(0.44-0.97) | 1.05(0.93-1.20) |
| 19 | 1.66(0.96-2.86) | 0.84(0.65-1.07) | 1.24(0.89-1.72) | 1.15(1.01-1.30) |
| 18 | 1.11(0.73-1.70) | 1.06(0.82-1.36) | 1.35(0.95-1.91) | 1.17(1.04-1.31) |
| 17 | 1.41(0.95-2.10) | 0.87(0.66-1.14) | 1.30(0.93-1.83) | 1.05(0.92-1.18) |
| 16 | 0.96(0.62-1.48) | 0.70(0.54-0.90) | 1.42(1.03-1.97) | 1.02(0.91-1.16) |
| 15 | 1.57(1.04-2.36) | 0.87(0.66-1.14) | 1.65(1.20-2.26) | 1.16(1.03-1.32) |
| 14 | 1.49(1.05-2.11) | 0.87(0.66-1.14) | 1.67(1.24-2.24) | 1.14(1.01-1.29) |
| 13 | 1.61(1.07-2.42) | 0.98(0.76-1.27) | 1.01(0.72-1.42) | 1.17(1.03-1.32) |
| 12 | 1.10(0.73-1.68) | 0.96(0.74-1.23) | 1.17(0.85-1.62) | 1.07(0.95-1.21) |
| 11 | 1.42(0.97-2.08) | 0.98(0.73-1.31) | 1.49(1.12-2.00) | 1.18(1.05-1.32) |
| 10 | 1.13(0.77-1.66) | 1.02(0.80-1.31) | 1.32(0.97-1.78) | 1.19(1.05-1.34) |
| 9 | 2.03(1.33-3.12) | 0.98(0.73-1.31) | 1.57(1.16-2.12) | 1.32(1.16-1.29) |
| 8 | 1.36(0.96-1.92) | 1.11(0.86-1.45) | 2.12(1.60-2.81) | 1.29(1.15-1.44) |
| 7 | 1.59(1.10-2.31) | 1.10(0.84-1.42) | 1.74(1.26-2.41) | 1.32(1.17-1.48) |
| 6 | 2.23(1.50-3.31) | 0.72(0.54-0.95) | 2.15(1.60-2.89) | 1.44(1.28-1.61) |
| 5 | 1.87(1.31-2.67) | 0.78(0.59-1.02) | 1.93(1.43-2.60) | 1.33(1.20-1.49) |
| 4 | 2.35(2.67-3.30) | 0,83(0.62-1.11) | 2.26(1.66-3.07) | 1.69(1.51-1.89) |
| 3 | 4.06(3.02-5.46) | 1.05(0.79-1.39) | 2.94(2.27-3.82) | 2.02(1.82-2.25) |
| 2 | 9.07(6.88-11.95) | 1.52(1.19-1.94) | 3.63(2.84-4.64) | 3.24(2.94-3.57) |
| 1 | 23.13(19.46-27.48) | 1.65(1.32-2.08) | 5.50(4.42-6.84) | 6.13(5.68-6.60) |
