# Supplementary material for: Primary Care Use before Cancer Diagnosis in Adolescents and Young Adults – A Nationwide Register Study
Source: PLoS One. 2016 May 20;11(5):e0155933. doi: 10.1371/journal.pone.0155933 (PMC4874574; doi:10.1371/journal.pone.0155933)
Supplement: S3 Table — (DOCX) [file pone.0155933.s003.docx]

**S3.** *Incidence rate ratios (IRR) for psychometric tests in primary care with 95% confidence intervals for central nervous system tumour and the total group two years before diagnosis (index date)*

|  | **Central nervous system tumour** | **Total** |
| --- | --- | --- |
| **Months before diagnosis** | IRR (95%CI) | IRR (95%CI) |
| 24 | 0.98(0.27-3.58) | 1.02(0.63-1.65) |
| 23 | 4.53(1.52-13.49) | 1.16(0.68-1.97) |
| 22 | 1.92(0.57-6.49) | 0.88(0.52-1.489 |
| 21 | 1.48(0.32-6.83) | 1.17(0.73-1.87) |
| 20 | 0.39(0.05-2.86) | 0.88(0.52-1.48) |
| 19 | 1.35(0.31-5.86) | 0.89(0.53-1.51) |
| 18 | 1.99(0.51-5.86) | 1.23(0.75-2.01) |
| 17 | 0.46(0.06-3.44) | 1.13(0.70-1.83) |
| 16 | 1.35(0.40-4.58) | 1.16(0.77-1.76) |
| 15 | 0.68(0.15-2.98) | 1.02(0.59-1.76) |
| 14 | 1.77(0.59-5.26) | 1.43(0.92-2.24) |
| 13 | 3.68(1.40-9.64) | 1.14(0.72-1.82) |
| 12 | 0.85(0.20-3.64) | 0.91(0-57-1.45) |
| 11 | 0.86(0.26-2.77) | 0.58(0.34-0.99) |
| 10 | 0.88(0.19-3.94) | 0.79(0.47-1.34) |
| 9 | 1.77(0.52-5.98) | 0.74(0.44-1.25) |
| 8 | 2.54(1.14-5.68) | 1.08(0.72-1.62) |
| 7 | 1.18(0.31-4.56) | 1.04(0.68-1.59) |
| 6 | 3.14(1.55-6.39) | 1.32(0.92-1.91) |
| 5 | 2.07(0.84-5.13) | 0.82(0.53-1.27) |
| 4 | 2.18(0.89-5.35) | 1.15(0.77-1.71) |
| 3 | 2.58(1.08-6.15) | 1.04(0.70-1.53) |
| 2 | 2.86(1.09-7.499 | 1.16(0.78-1.72) |
| 1 | 3.50(1.49-8.24) | 1.49(1.05-2.10) |
